# Supplementary material for: Atom-level interaction design between amines and support for achieving efficient and stable CO2 capture
Source: Nat Commun. 2024 Jun 13;15:5068. doi: 10.1038/s41467-024-48994-8 (PMC11176289; doi:10.1038/s41467-024-48994-8)
Supplement: Supplementary file 1 — Supplementary Information [file 41467_2024_48994_MOESM1_ESM.pdf]

Supplementary Information

Atom-level interaction design between amines and support for achieving efficient and stable CO_2 capture

Xin Sun et al

This file includes:

Supplementary Figures 1 to 11

Supplementary Table 1

Supplementary Notes 1 to 2

Supplementary References 1 to 14

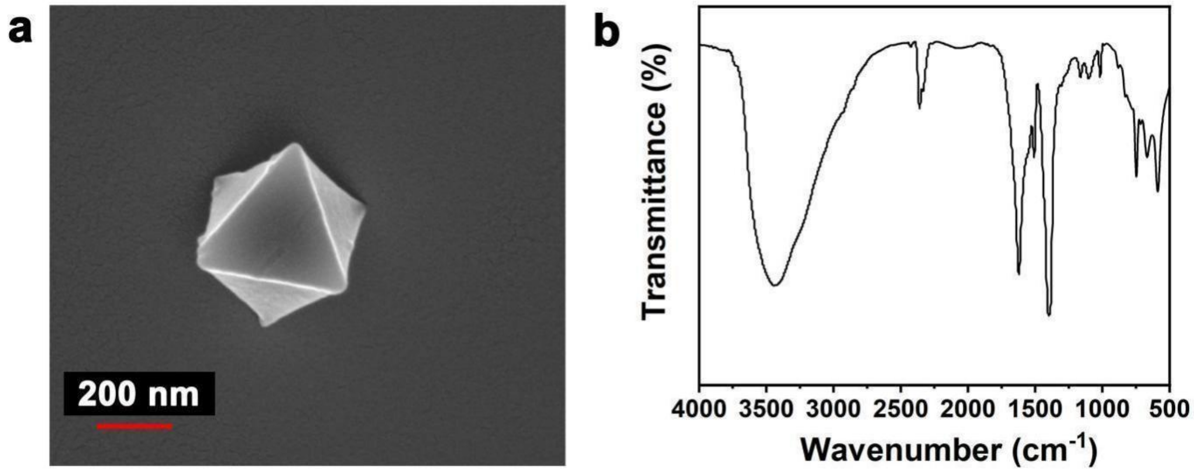

Supplementary Figure 1 | The characters of MIL-101(Cr) support. a SEM image of MIL-101(Cr) support. **b** FT-IR spectrum.

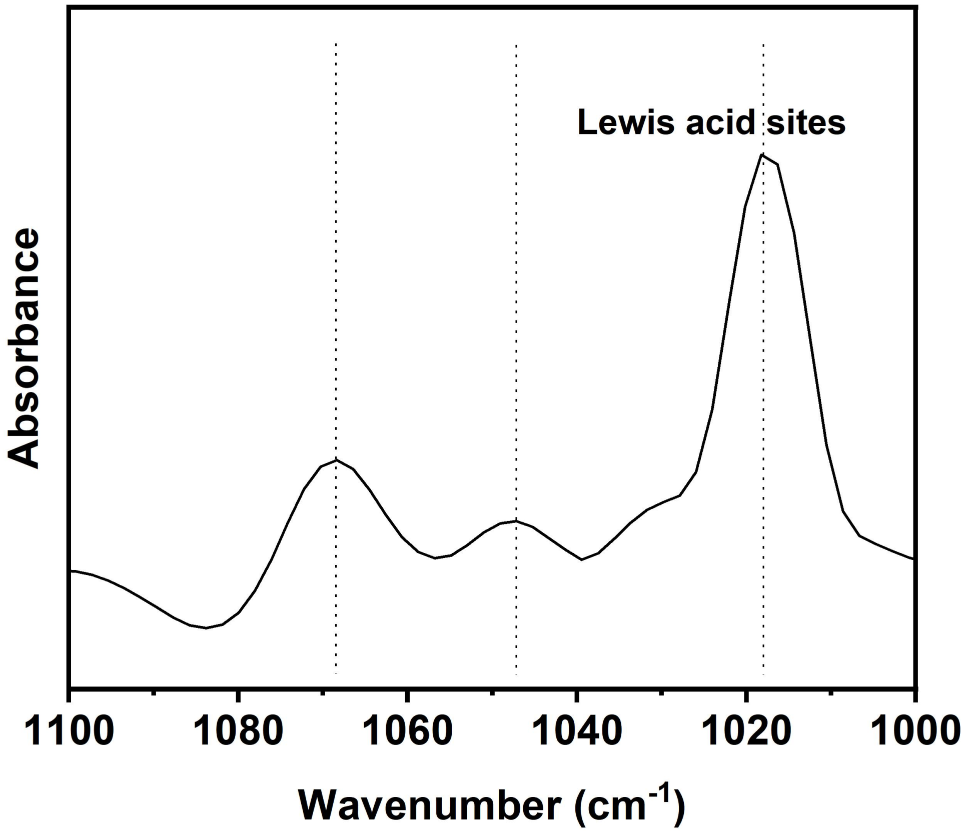

Supplementary Figure 2 | The identify of Lewis acid sites in MIL-101(Cr) support. FT-IR spectra observed after adsorption of pyridine for MIL-101(Cr) followed by vacuum treatment at 473 K.

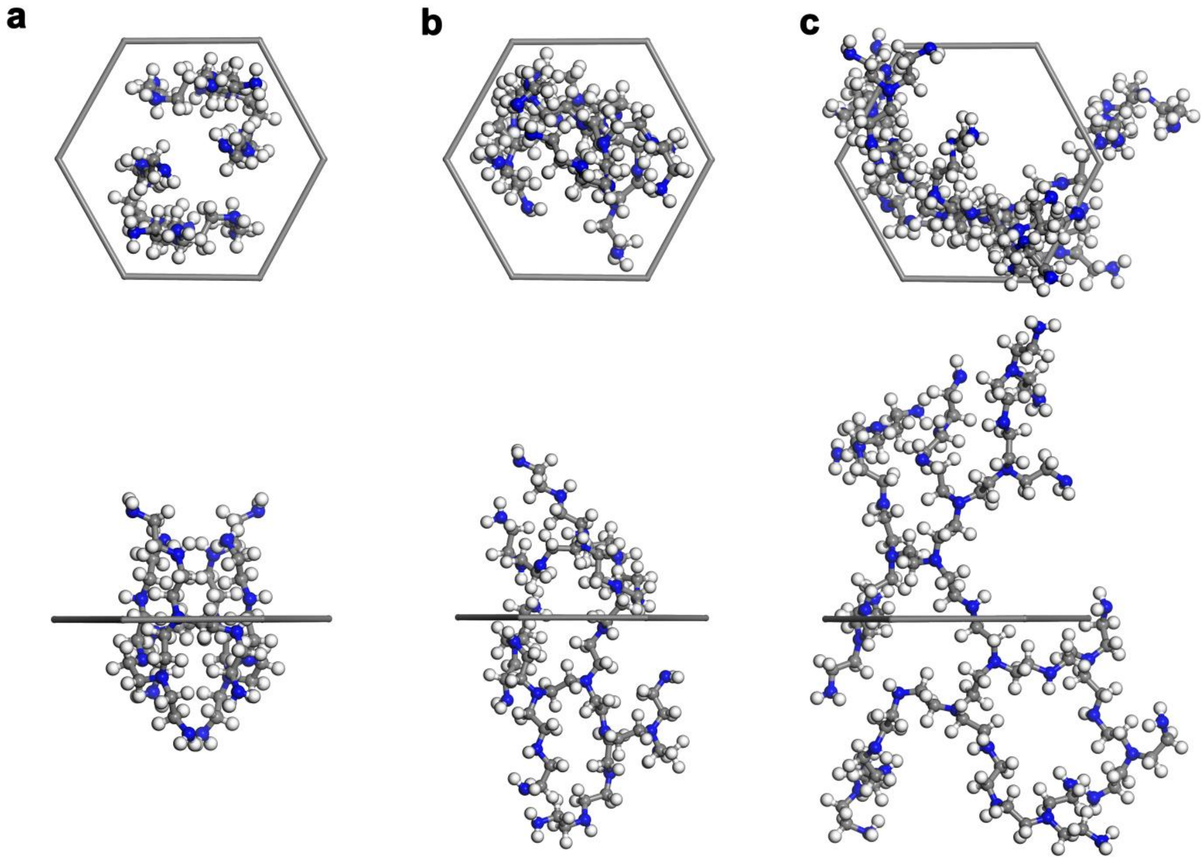

Supplementary Figure 3 | The scenarios of different molecular weights of PEI passing through the hexagonal windows in MIL-101(Cr) from the perspective of molecular size. **a** simultaneous passage of two PEI-600 molecules. **b** Passage of only one PEI-1200 molecule precisely. **c** The clogging of PEI-1800. Gray, blue, and white spheres represent C, N, and H, respectively, the gray hexagon represent the window pore of cages in MIL-101(Cr).

Supplementary Note 1 | The model of different molecular weights PEI

The atomic model parameter and binding energies were performed using the Forcite module of Materials Studio software package. During the geometry optimizations, all the atom position were allowed to relax. The geometry optimization convergence thresholds for energy change, maximum force, and maximum displacement between optimization cycles were chosen to be 10^{-4} kcal/mol, 0.005 kcal/mol /Å, and 0.005 Å, respectively. The charge is Forcefield assigned, the van der Waals and electrostatic summation methods are atom based. The total potential energy calculated based on COMPASS force field is composed of three main components, including bond energy terms, non-bond energy terms and cross energy terms, as described by Equations (1) − (4)[1,2]

$$E_{potential} = E_{bond} + E_{non-bond} + E_{cross} \quad (1)$$

$$E_{bond} = E_b + E_\theta + E_\varphi + E_{inv} \quad (2)$$

$$E_{non-bond} = E_{vdW} + E_{elec} \quad (3)$$

$$E_{cross} = E_{b,b'} + E_{\theta,\theta'} + E_{b,\varphi} + E_{b,\theta} + E_{\theta,\varphi} + E_{\theta,\theta',\varphi} \quad (4)$$

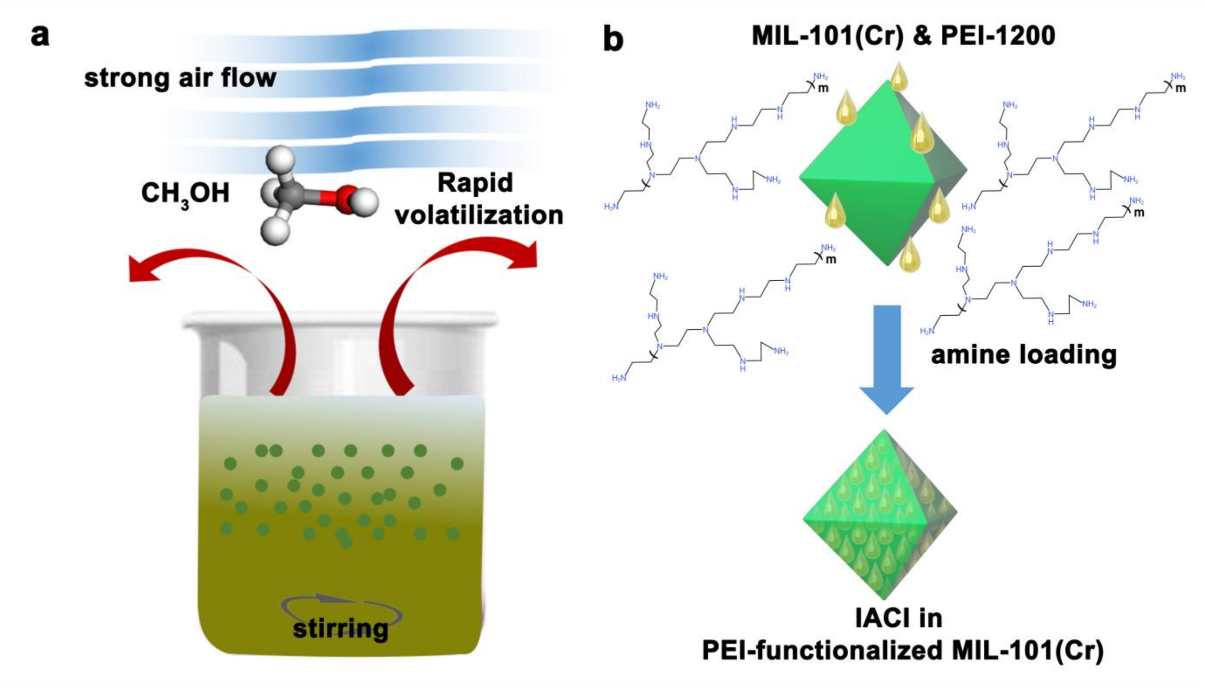

Supplementary Figure 4 | The synthesis process of impregnating amine into crystal internalization (IACI) in PEI-functionalized MIL-101(Cr). a Schematic diagram of actual impregnation operation. **b** The formation of IACI in PEI-functionalized MIL-101(Cr).

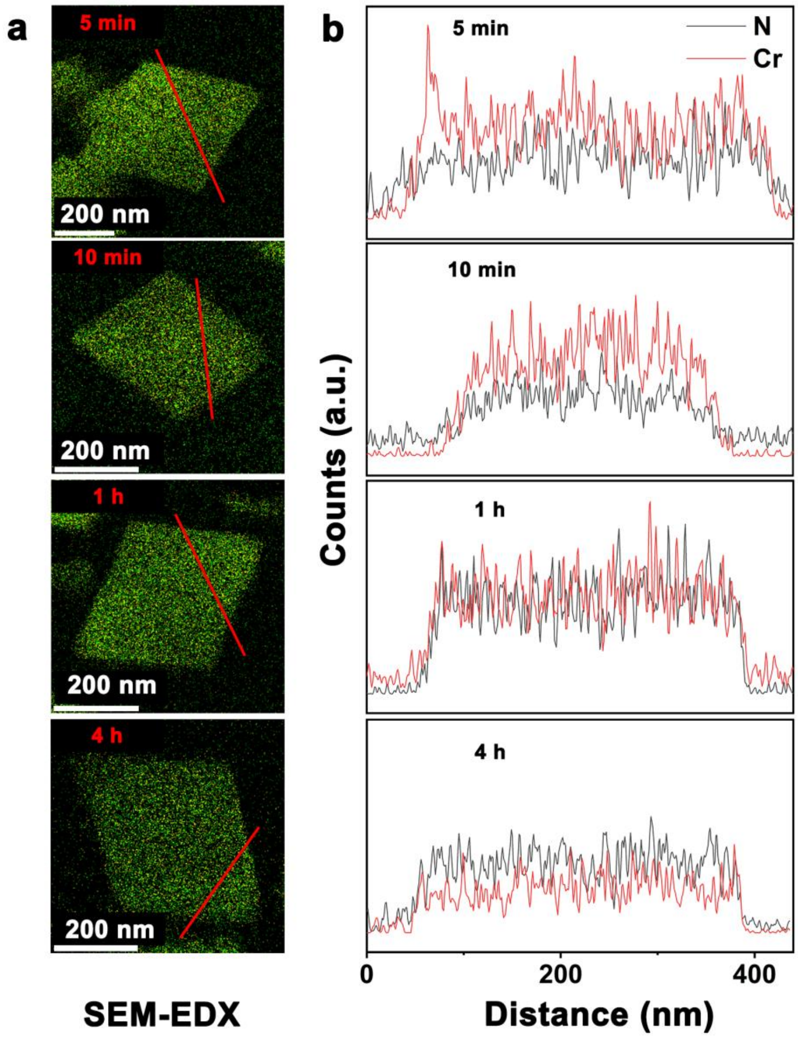

Supplementary Figure 5 | The element distribution of N and Cr in the ultrathin cut from PEI-functionalized MIL-101(Cr) under different impregnation time. a The EDX line scan sites. **b** The N and Cr change with position.

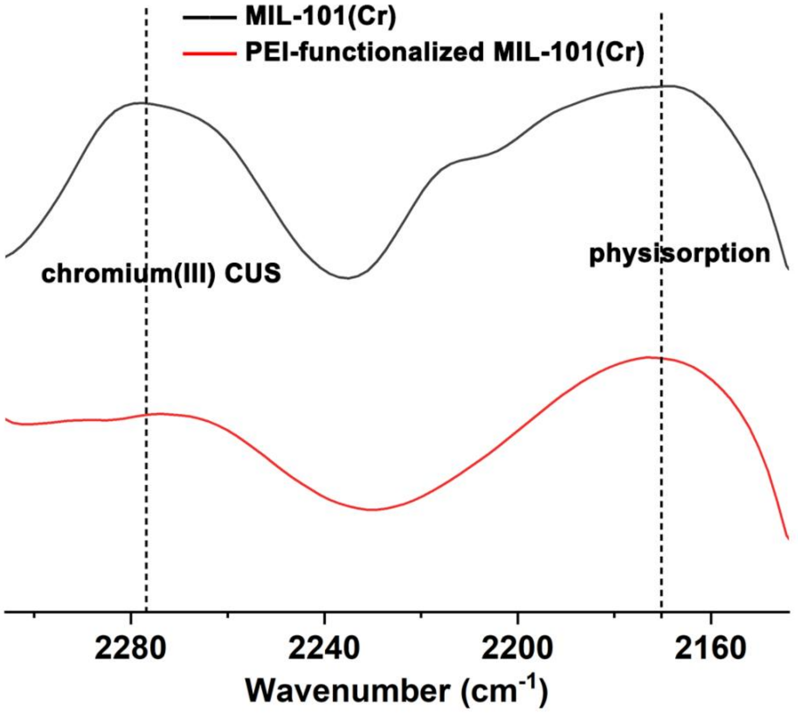

Supplementary Figure 6 | FT-IR spectra of the MIL-101(Cr) and PEI-functionalized MIL-101(Cr) characterization. FT-IR spectra recorded at 100 K of MIL-101(Cr) and PEI-functionalized MIL-101(Cr) after introduction of an equilibrium pressure (200 Pa) of CO_2.

Supplementary Note 2 | The calculation for the consumption comparison

To investigate the cost-effective properties of PEI-functionalized MIL-101(Cr) for CO_2 capture, we compared the amine consumption, energy consumption, and cycle time — the primary costs involved in CO_2 capture. Here are the details:

Amine consumption. In the CO_2 capture process using amine-functionalized adsorbents, amine deactivation results in significant consumption due to the high cost and non-renewable nature of amines. Based on industrial process observations[3], we assumed that, on average, each unit mass of amine-functionalized adsorbents would be discarded when the adsorption capacity decreases by half to maintain carbon capture efficiency. First, we calculated the number of cycles (N) and the corresponding adsorption capacity (X_N) based on the initial CO_2 uptake (X) and the average inactivation efficiency per cycle (η) using Equation (5). Then, when X_N is less than half of X, we obtained the value (n), which represents the estimated number of cycles for practical application of these amine-functionalized adsorbents. Based on n, the total CO_2 capture capacity of the adsorbents (X_{all}) is calculated using Equation (6). As a result, we calculated the amine consumption (C_{amine}) using Equation (7).

Energy consumption. Diffusion resistance during CO_2 adsorption often requires heating the adsorbed gas, and estimating the energy associated with this heating is obscure due to variations in gas composition and initial temperature. Therefore, we only considered the energy required for desorption as the energy consumption for the entire cycle. It is important to note that the energy consumption of the adsorbent requiring heating during the adsorption process should be greater than this value.

Cycle time. In practical carbon capture processes, numerous equipment components operate to maintain the entire reaction system. A shorter cycle time implies lower additional energy input and higher carbon capture efficiency per unit time, which may vary depending on specific process parameters[4]. Furthermore, disregarding minor and uncontrollable factors such as material replacement and equipment maintenance, the cycle time (t_{cycle}) can be described by Equation (8).

$$X_N = X_a \times (1-\eta)^N \quad (5)$$

$$X_{all} = \sum_{k=1}^{k=n} X_k \quad (6)$$

$$C_{amine} = (m_{adsorbent} \times \text{wt.\%})/X_{all} \quad (7)$$

$$t_{cycle} = t_a + t_d \quad (8)$$

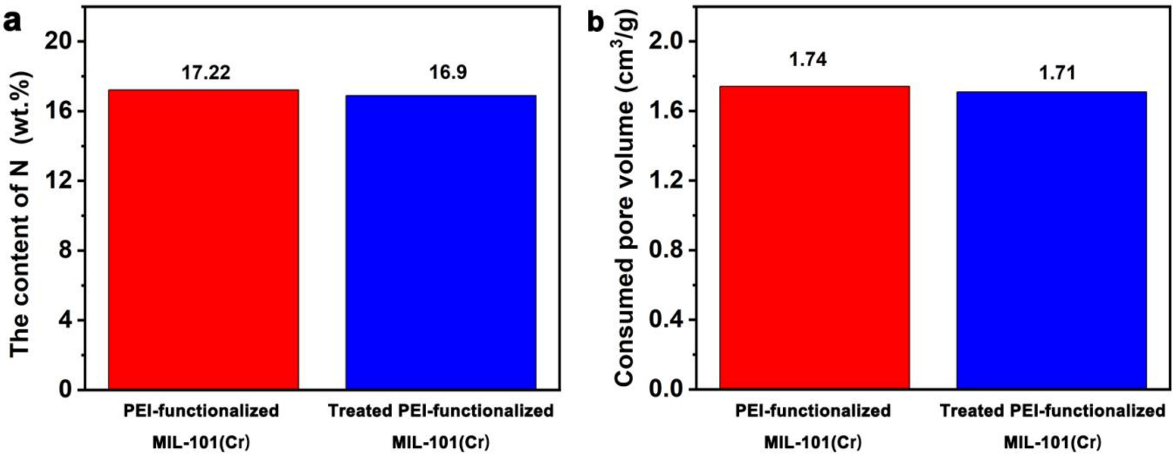

Supplementary Figure 7 | Characteristic change of PEI-functionalized MIL-101(Cr) adsorbent before and after washing. **a** The N content change in PEI-functionalized MIL-101(Cr) adsorbent. **b** Consumed pore volume change of PEI-functionalized MIL-101(Cr) adsorbent.

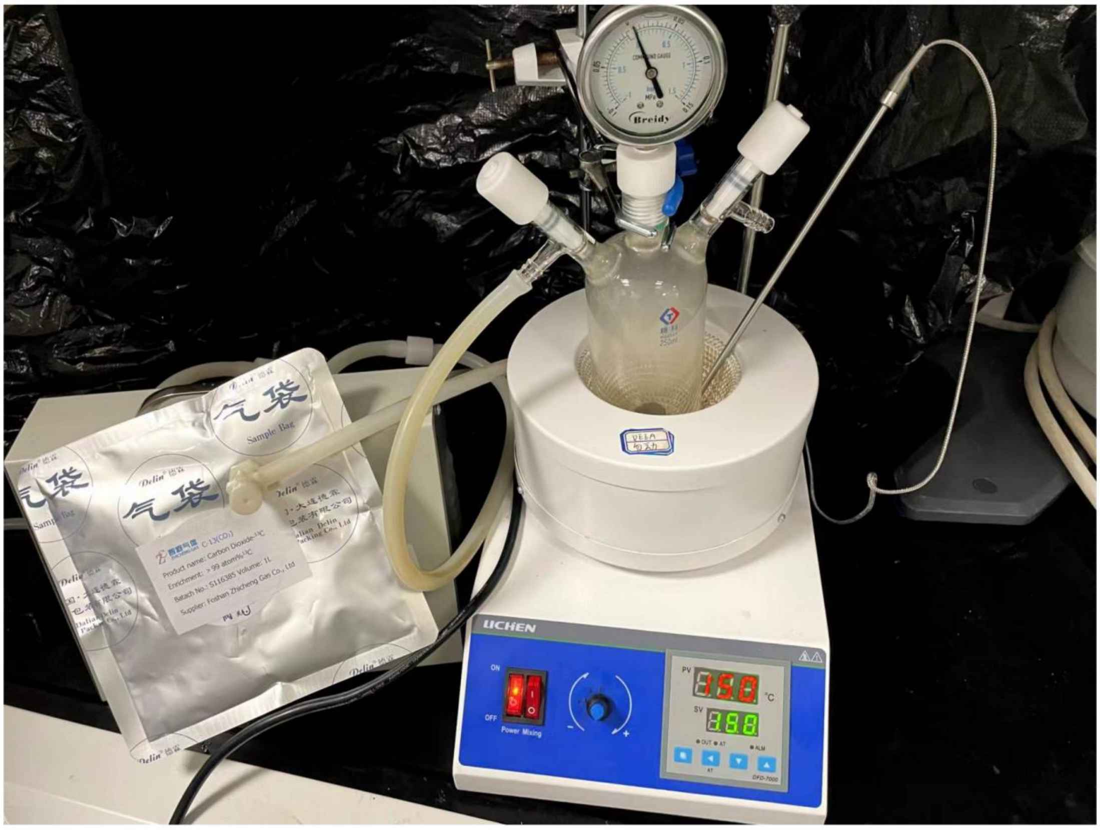

Supplementary Figure 8 | Test rig for checking the urea distribution in PEI-functionalized MIL(Cr) adsorbent (using a $^{13}CO_2$ gas).

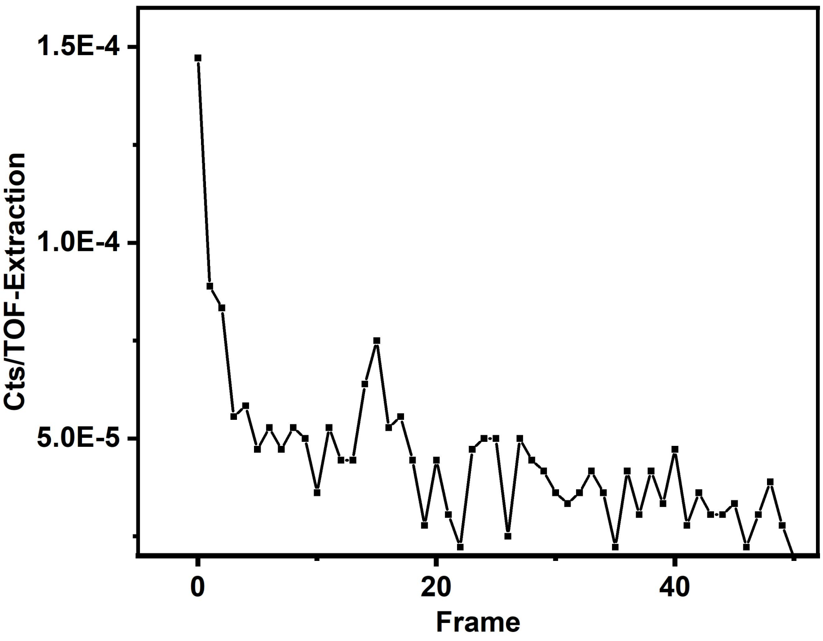

Supplementary Figure 9 | TOF-SIMS of ^{13}C secondary ion (urea distribution) depth profiles through an originally 60 frames.

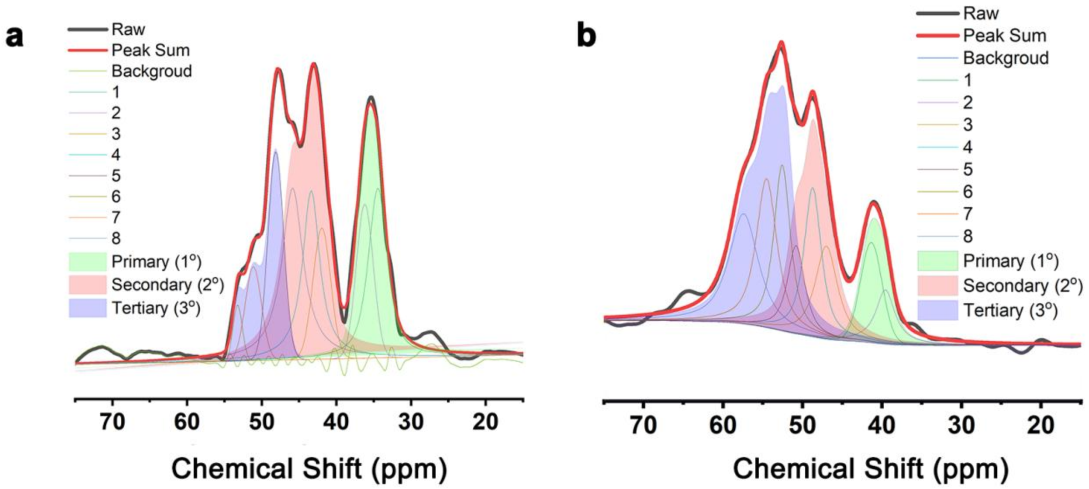

Supplementary Figure 10 | The solid-phase ^{13}C NMR spectra of **a** 55%PEI@SiO_2 and **b** PEI-functionalized MIL-101(Cr) adsorbents.

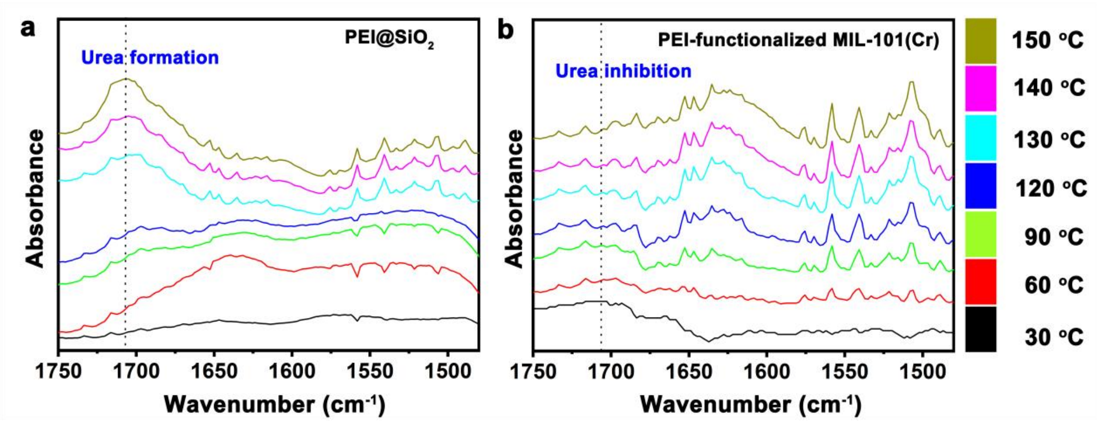

Supplementary Figure 11 | *In situ* FTIR spectra of **a** 55%PEI@SiO_2 and **b** PEI-functionalized MIL-101(Cr) adsorbents as a function of adsorption temperature at humid (~5% H_2O) CO_2 stream with the activated sample as the background. the PEI@SiO_2 adsorbent exhibited a significant peak increase at 1706 cm^{-1}, indicating the formation of urea.[5] On the other hand, no such substance formation was observed for the PEI-functionalized MIL-101(Cr), suggesting its pronounced resistance to urea under humid conditions.

Supplementary Table 1 | Comparison of CO_2 capture properties between PEI-functionalized MIL-101(Cr) and previously reported amine-functionalized adsorbents. Some detailed characteristics can be found in the ref. 6-8.

Adsorbents		CO_2 Capture		Characteristics		
Type	Materials	Adsorption temperature (°C)	CO_2 uptake (mmol/g)	Advantages	Disadvantages	ref.
activeted carbon (AC)	KOH-treated AC	30	0.84	water resistance; high stable; low recovery energy	low capacity and selective	9
zeolite	NaX	40	2.10	high stable; tunable structure	humid sensitive; low capacity under low pressure	10
MOFs	Mg_2(dobdc)	40	4.95	high capacity; highly tunable structure	humid sensitive; low stability	11
alkali based	CaO	650	9.32	technological maturity; high capacity; water resistance	high recovery energy and temperature input;	12
liquid amine	MEA&DMA2P	30	3.06	high capacity; water resistance; technological maturity;	oxidative degradation; expel toxic volatiles	13
typical solid support amine	PEI@SiO_2	90	3.0	high capacity; water resistance	oxidative degradation; low diffusion kinetics	14
IACI	PEI-functionalized MIL-101(Cr)	5	4.0	high capacity; water resistance; high stable and diffusion kinetics	technological immaturity	This study

Supplementary References

1. Sharma, S., Chandra, R., Kumar, P. & Kumar, N. Thermo-mechanical characterization of multi-walled carbon nanotube reinforced polycarbonate composites: A molecular dynamics approach. *Comptes Rendus Mécanique*. **343**, 371-396 (2015).
2. Qi, G., Fu, L. & Giannelis, E. P. Sponges with covalently tethered amines for high-efficiency carbon capture. *Nat. Commun.* **5**, (2014).
3. Patel, H. A., Byun, J. & Yavuz, C. T. Carbon dioxide capture adsorbents: Chemistry and methods. *Chemsuschem*. **10**, 1303-1317 (2017).
4. Keith, D. W., Holmes, G., Angelo, D. S. & Heidel, K. A process for capturing CO_2 from the atmosphere. *Joule*. **2**, 1573-1594 (2018).
5. Li, K. et al. Research on Urea Linkages Formation of Amine Functional Adsorbents During CO_2 Capture Process: Two Key Factors Analysis, Temperature and Moisture. *The Journal of Physical Chemistry C*. **120**, 25892-25902 (2016).
6. Ozkan, M., Akhavi, A., Coley, W. C., Shang, R. & Ma, Y. Progress in carbon dioxide capture materials for deep decarbonization. *Chem*. **8**, 141-173 (2022).
7. Fu, D. & Davis, M. E. Carbon dioxide capture with zeotype materials. *Chem. Soc. Rev.* **51**, 9340-9370 (2022).
8. Kolle, J. M., Fayaz, M. & Sayari, A. Understanding the Effect of Water on CO_2 Adsorption. *Chem. Rev.* **121**, 7280-7345 (2021).
9. Liu, L., Jin, S., Park, Y., Park, Y. C. & Lee, C. Sorption Equilibria and Kinetics of CO_2, N_2, and H_2O on KOH-Treated Activated Carbon. *Ind. Eng. Chem. Res.* **57**, 17218-17225 (2018).
10. Kim, C., Cho, H. S., Chang, S., Cho, S. J. & Choi, M. An ethylenediamine-grafted Y zeolite: a highly regenerable carbon dioxide adsorbent via temperature swing adsorption without urea formation. *Energy Environ. Sci.* **9**, 1803-1811 (2016).
11. Mason, J. A. et al. Application of a High-Throughput Analyzer in Evaluating Solid Adsorbents for Post-Combustion Carbon Capture via Multicomponent Adsorption of CO_2, N_2, and H_2O. *J. Am. Chem. Soc.* **137**, 4787-4803 (2015).
12. Han, R., Gao, J., Wei, S., Su, Y. & Qin, Y. Development of highly effective CaO@Al_2O_3 with hierarchical architecture CO_2 sorbentsvia a scalable limited-space chemical vapor deposition technique. *J. Mater. Chem. A*. **6**, 3462-3470 (2018).
13. Wang, L., Tian, X., Fu, D., Du, X. & Ye, J. Experimental investigation on CO2 absorption capacity and viscosity for high concentrated 1-dimethylamino-2-propanol – monoethanolamine aqueous blends. *The Journal of Chemical Thermodynamics*. **139**, 105865 (2019).
14. Meng, Y. et al. Comprehensive study of CO_2 capture performance under a wide temperature range using polyethyleneimine-modified adsorbents. *J. CO_2 Util.* **27**, 89-98 (2018).
